# How Do Video Games Elicit Guilt in Players? Linking Character Morality to Guilt Through a Mediation Analysis

**DOI:** 10.3389/fpsyg.2021.666518

**Published:** 2021-06-22

**Authors:** Changhyun Ahn, Matthew Grizzard, Seyoung Lee

**Affiliations:** ^1^Health and New Media Research Institute, Hallym University, Chuncheon, South Korea; ^2^School of Communication, Ohio State University, Columbus, OH, United States; ^3^Department of Media and Communication, Sungkyunkwan University, Seoul, South Korea

**Keywords:** narrative, morality, self-serving bias, attribution, guilt

## Abstract

Research has consistently found that committing immoral actions in video games is capable of eliciting feelings of guilt in players. This study aimed to investigate the mediating role of theoretically-relevant psychological mechanisms: Perceived morality of the player-controlled character and self-attribution of virtual behavior. Based in psychological and communication theory, we derived a model that links these variables to character portrayal and guilt. A between-subjects experiment manipulated the portrayal of the player-controlled character (immoral vs. moral) and measured the mediating variables and self-reported guilt. The hypothesized model was tested using a path model. Data were generally consistent with hypotheses. Controlling an immoral character reduced perceived character morality. Perceived character morality positively predicted self-attribution of character behavior and negatively predicted guilt. Self-attribution positively predicted guilt but self-attribution and perceived character morality did not interact. Our findings suggest novel directions for continued research into how game features elicit emotional responses in players.

## Introduction

Engaging in immoral actions in virtual settings, such as video games can lead players to feel guilt (Hartmann and Vorderer, 2010; Hartmann et al., 2010; Lin, 2011; Weaver and Lewis, 2012; Grizzard et al., 2014, 2017; Mahood and Hanus, 2017). Guilt is typically defined as a self-conscious emotion, which requires the activation of higher-level cognitive appraisal processes (Tangney et al., 2007; de Hooge, 2008). Guilt results when a person has violated a moral or social norm and attributes responsibility for the violation to the self (i.e., an internal attribution; e.g., I did this because I wanted to; Weiner, 2018). Absent a moral violation, guilt would not be expected to occur. Moreover, external attributions (e.g., “I did this because I was forced to”) would be expected to reduce feelings of responsibility and guilt (see O'Donnell, 2005; Klimmt et al., 2006; Tangney et al., 2007; de Hooge, 2008; Hartmann and Vorderer, 2010; Hartmann et al., 2010; Weiner, 2018).

Virtual actions, by definition, lack real-world consequences. Moreover, games and their rule-based systems provide players with an external factor other than the self to which they might attribute their behaviors. Such external attributions should prohibit the elicitation of guilt (see Bartel, 2015). Yet, past research (Hartmann and Vorderer, 2010; Hartmann et al., 2010; Lin, 2011; Weaver and Lewis, 2012; Grizzard et al., 2014, 2017; Mahood and Hanus, 2017) that finds a relationship between such actions and guilt suggests that some players (a) perceive their virtual actions to be morally consequential, and (b) attribute responsibility for that consequentiality to themselves. Specific mechanisms that explain these phenemona have been theorized but remain untested. Thus, the current paper aims to bridge the gap by examining the role of character portrayal and perceptions of a character's morality/immorality as antecedents of guilt. Specifically, we test a causal model that links features of video game play (e.g., the character's narrative portrayal) to guilt through perceptions of the character's morality. We simultaneously examine how character portrayal influences self-attribution of character behavior and any moderating impact this would have on the generation of guilt. Thus, the specific model tested in the current study unites psychological understandings of guilt elicitation (i.e., immoral behaviors must be attributed to the self in order to elicit guilt) with common features of video game play (i.e., the experience of role-playing as a specific character). It also tests current theory regarding the interaction between character morality and player perceptions (see Bartel, 2015). In so doing, the current paper and its findings provide (a) empirical evidence for psychological theories of guilt and game studies theories of video game experience and (b) demonstrates a method to reliably alter player's perceptions of their in-game experience (i.e., the manipulation of a player character's portrayal).

## Character Portrayal, Guilt, and Perceived Morality of Character

Several variables seem capable of eliciting guilt in games, including the attributes of the player-character (i.e., the character/avatar controlled by the player), the morality of virtual behaviors, and the attributes of non-player-characters (NPCs; i.e., those virtual agents who interact with the player-character). Controlling a player-character who is immoral elicits higher levels of guilt than controlling a player-characters who is moral (Hartmann and Vorderer, 2010; Hartmann et al., 2010; Mahood and Hanus, 2017). Committing less moral behaviors results in greater guilt than more moral behaviors (Mahood and Hanus, 2017). Finally, committing aggression against humanized NPCs results in greater guilt than committing aggression against dehumanized NPCs (Lin, 2011). These findings suggest that factors which positively or negatively influence the perceived morality of an action in the “real world” have similar effects in the virtual world (O'Donnell, 2005; Klimmt et al., 2006). The most common manipulation from past research seems to be attributes of the player-character, and so for the current paper, we focus on this manipulation and hypothesize the following:

Hypothesis 1: Character portrayal (manipulated moral vs. immoral) will influence perceptions of the character's morality.

Hypothesis 2: Perceived character morality will negatively predict guilt.

Hypothesis 3: Perceived character morality will mediate the relationship between character portrayal and guilt.

## Perceived Morality of Character, Self-Attribution, and Guilt

The attribution literature defines attributions in terms of their internal and external nature (Heider, 1958; Kelley, 1967; Weiner, 2018). Internal attributions are those that relate to the purposeful desires and motivations of an individual, whereas external attributions are those that relate to the environment that an individual finds themselves in. As stated earlier, recognition that one's behavior has violated a moral or social norm is insufficient to elicit guilt. Guilt responses require an internal attribution of the cause of the behavior and a perception of one's actions as volitional.

External attributions reduce feelings of guilt (see Tangney et al., 2007; de Hooge, 2008; Weiner, 2018), and video games have several features that should allow players to make external attributions for their immoral behaviors (Bartel, 2015). First, narrative video games place players into an artificial world governed by a rule-based system, which should allow a player to make an external attribution for their behavior (Klimmt et al., 2006). Second, players' in-game behaviors are mediated through the character or avatar they are controlling. The perception of an avatar as a separate entity from oneself (see Banks, 2015; Grizzard and Ahn, 2017) should allow for an external attribution of behavior. Finally, a general bias related to internal and external attributions—the *self-serving bias* (Miller and Ross, 1975)—should also reduce guilt. This bias relates to humans attributing desirable outcomes to themselves and undesirable outcomes to external factors (see Miller and Ross, 1975; see also for update, Weiner, 2018). Conceptualizing virtual immoral actions as undesirable suggests that internal attributions would be reduced for players who control an immoral character and internal attributions would be enhanced for players who control a moral character.

Hypothesis 4: Perceived character morality will positively predict self-attributions.

Combining self-attributions with past findings regarding the ability of virtual immoral actions to elicit guilt (Hartmann and Vorderer, 2010; Hartmann et al., 2010; Mahood and Hanus, 2017) suggests a potential moderation effect. Bartel (2015) argued that players will only feel guilt for their immoral virtual behaviors if they attribute the cause of such behaviors to the self. This proposition suggests that the effect of perceived character morality on guilt will be moderated by self-attribution, such that self-attributions increase the strength of the relationship.

Hypothesis 5: Self-attribution will moderate the effect of perceived character morality on guilt.

## Method

### Participants

Study participants (*N* = 101) were recruited to from communication classes at a large, public university in the northeastern United States (40 women, 61 men, *M*_*age*_ = 19.84, *SD* = 3.08), and received extra credit for their participation. Participants were treated in accordance with the ethical standards of the American Psychological Association and all procedures were evaluated by an institutional review board.

### Design and Procedure

Participants were randomly assigned to play a video game as a character with either an immoral (*n* = 52) or a moral (*n* = 49) character portrayal. Participants first read a mock webpage that manipulated the morality of the character. They then played the game, and following play were asked to rate the key variables using self-report measures.

### Stimuli

*Heavy Rain*, a PlayStation 3/4 game about catching a serial killer, was selected as the stimulus. It was selected because (1) it is an interactive narrative game which allows for manipulation of the player-character's morality and (2) Scott Shelby, one of the playable characters in this game is a morally ambiguous character who can be easily manipulated to appear good or evil. A mock webpage was used to manipulate Shelby's portrayal. In the moral character portrayal, Shelby was described as a private detective hired by a victim's family to stop the Origami Killer. In the immoral character portrayal, Shelby was described as the Origami Killer who was simply pretending to be a private detective in order to cover his tracks. The different portrayals were controlled for length and structure.

Participants completed the game chapter “Sleazy Place.” In the chapter, Shelby interviews the mother of one of the killer's victims. Following questioning, the mother begins to cry. A result of our manipulation is that both conditions played the exact same game with the same characters and in-game results, with the only difference being the perceived motivations of the character. Gameplay lasted ~7 min. The stimulus was played through a PlayStation 4 with 32-inch VIZIO 1,080 p 120 Hz (native) screen. Participants were seated approximately half a meter from the screen. The default sound system from the television was used.

#### Permission to Reuse and Copyright

Figures, tables, and images will be published under a Creative Commons CC-BY license and permission must be obtained for use of copyrighted material from other sources (including re-published/adapted/modified/partial figures and images from the internet). It is the responsibility of the authors to acquire the licenses, to follow any citation instructions requested by third-party rights holders, and cover any supplementary charges.

### Measures

*Character moral portrayal* was dummy-coded (1 = immoral, 0 = moral).

*Perceived morality of game character* was measured with a single item: “Is Scott a good guy or a bad guy?”. The item was rated on a 7-point semantic-differential scale ranging from 1 *bad* to 7 *good*. An independent-samples *t*-test indicated that participants in the immoral condition perceived their character to be less moral (*M* = 2.17, *SD* = 1.49) than participants in the moral condition (*M* = 5.18, *SD* = 1.11), *t*(99) = −11.45, *p* < 0.001. The effect size associated with this difference is large (Cohen's *d* = −2.29) and suggests that the manipulation was powerful for eliciting differential perceptions of the player character's morality.

*Self-attributions of virtual behavior* was measured using 7-point Likert-type responses to four items: “The actions my character committed represent me as a person,” “The actions I committed as Scott Shelby were an expression of my true inner feelings, attitudes, and beliefs,” “I felt in control of Scott Shelby's actions,” and “I felt personally responsible for the virtual actions that I committed in the game.” To assess the statistical validity of the scales, we used confirmatory factor analysis (CFA). The five self-attribution items were tested in a CFA with a single latent factor. The model fit the data well, χ^2^(*df* = 2) = 3.73, *p* = 0.16, CFI = 0.98, RMSEA = 0.09 (90% CI: 0.00,.24), SRMR = 0.05, and were averaged to create a composite. Internal consistency of the scale was judged to be acceptable as Cronbach's alpha approached 0.70 (α = 0.68) and McDonald's omega—a more robust estimate of internal consistency (see Hayes and Coutts, 2020) −0.70 (ω = 0.70).

*Guilt* was measured using a 6-item guilt scale with 11-point Likert-type response scale ranging from 0 = *not at all* to 10 = *extremely*. A CFA on the items resulted in an acceptable fit: χ^2^(*df* = 9) = 44.65, *p* < 0.001, CFI = 0.94, RMSEA = 0.20, SRMR = 0.04. We note that although RMSEA is elevated here, small *df* models can have artificially large RMSEAs (see Kenny et al., 2015, who recommend not calculating RMSEA for small *df* models). Given the other model fit indices (CFI, SRMR) suggest good to excellent fit, we created a composite by averaging across the six items (α = 0.95; ω = 0.95; Hartmann and Vorderer, 2010; Hartmann et al., 2010).

### Analysis

**Figure 1:**
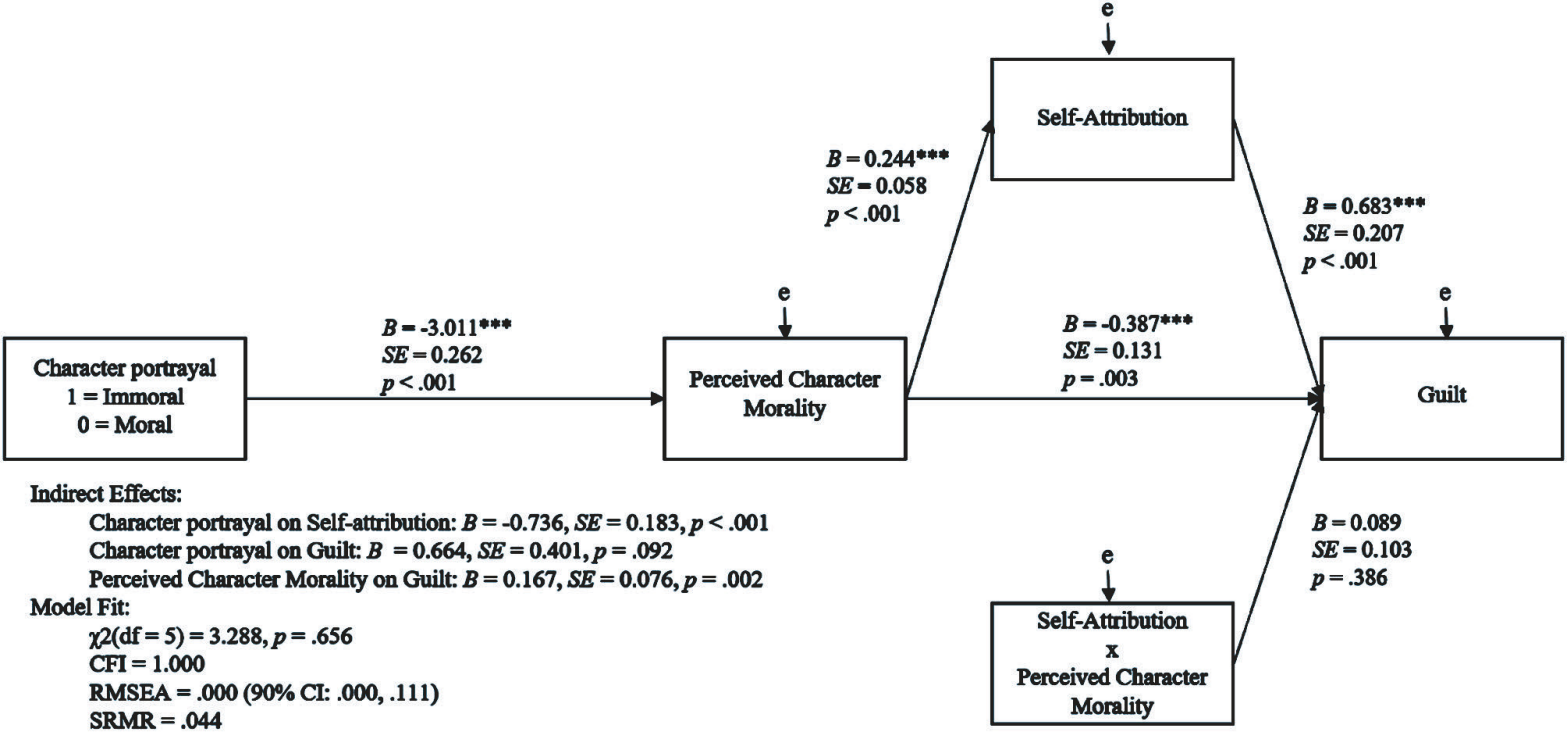
Path model examining the hypotheses of the current study (^***^p < 0.001).

We tested the hypotheses in a path model in AMOS using maximum likelihood estimation. Indirect effects were assessed through 5,000 bootstrap samples with bias-corrected 95% confidence intervals. The interaction term in the model was created after mean-centering the two variables that composed it. See Figure 1 for the model and the results of the model test.

## Results

### Hypotheses Testing

Data were consistent with Hypothesis 1. The character portrayal manipulation (dummy-coded 1 = immoral, 0 = moral) had a significant effect on perceived character morality. The player character was perceived as more moral when the moral character portrayal was associated with him.

Data were also consistent with Hypothesis 2. Perceived character morality had a negative effect on guilt. The more immoral the player character was perceived to be, the more guilt the player felt following game play.

Data were inconsistent with Hypothesis 3. The indirect effect of condition was non-significant (*p* = 0.092).

Data were consistent with Hypothesis 4. The more moral the player character was perceived to be, the more players attributed the character's behaviors to themselves. In addition, the indirect effect of condition on self-attribution was mediated through perceived character morality.

Data were inconsistent with Hypothesis 5. Although the direct path from self-attribution to guilt was significant, the interaction of self-attribution and perceived character morality was non-significant. This indicates that the more the players attributed the character's behavior to themselves the more guilt they felt regardless of the perceived morality of the character. It is important to note that the indirect effect of perceived character morality on guilt through self-attribution was significant. Together these findings suggest that perceptions of a character impact moral emotions mediated through self-attribution. One reason we may not have found a significant moderation could result from the narrative ending of the game play session. In both sessions, the player character causes the mother of a murder victim to recall a painful episode, which results in her crying. Our findings indicate that the more the participant attributed the actions of the character to themselves, the more guilt they felt regardless of the character's perceived morality. This finding makes sense when one considers that the ending was an undesirable and potentially guilt-inducing result that was magnified by the characters role (i.e., the significant results of Hypothesis 2) but not the attribution of the character's behavior to the self (i.e., the non-significant interaction term).

## Discussion

The goal of the current study was to examine psychological mechanisms through which immoral virtual actions influence guilt following video game play. Results suggest that a character's portrayal can have a significant impact on the perception of a character as moral and in turn the guilt elicited from immoral virtual actions. In addition, our findings demonstrate that a self-serving attributional bias can occur in virtual settings. Behaviors by a desirable virtual other are attributed to the self to a greater extent than behaviors by an undesirable virtual other. Our findings make several contributions to the literature on video game behaviors and the elicitation of guilt.

First, our findings begin to build and test a serial causal model of how player characters are interpreted and experienced by players. This model links video game attributes to player responses in a novel way. We found that players accurately perceive the morality of their player character and that these perceptions have direct impacts on guilt and the attribution of the character's behavior to the self. Future research should continue to explore these effects by manipulating aspects of the character's portrayal and examining other person perception attributes. For example, does controlling a character portrayed as powerful lead to feelings of competence for the player in the same way that controlling a character portrayed as immoral led to feelings of guilt? How might attributes of non-player characters impact these relationships? Would a player feel less guilty controlling an immoral character if NPCs were even less moral (see the character interdependence hypothesis; Grizzard et al., 2020)? These questions could be answered by applying our design and logic to other variables.

Second, our study provides a useful methodological observation. Our findings validate techniques for manipulating video game stimuli in a methodologically sound manner that maximizes internal validity and minimizes costs. Past studies (see Hartmann et al., 2010) have manipulated character portrayal by modifying a game's code. This approach is useful, but costly and time-consuming. We were able to achieve similar effects as this past work through simpler means: Creating a character biography that portrayed the character as moral or immoral. Our findings show that this type of exo-game manipulation (see Grizzard and Ahn, 2017) can be applied to some degree of success in a laboratory setting. By manipulating the character's backstory, we were able to induce variance in a theoretically meaningful way (a) without compromising internal validity by utilizing two separate games and (b) without having to engage in costly and time-consuming programming.

Our findings thus contribute to research on the emotions elicited by game play (see Grizzard and Francemone, 2018) by explicitly testing the implicit mechanisms described in previous research and theory (Klimmt et al., 2006; Hartmann et al., 2010; see Bartel, 2015). In addition, we provide nuance to the assumption that a player must attribute immoral actions to themselves to elicit guilt. This hypothesis provided by Bartel (2015) was partially consistent with our results. We found a significant effect of self-attribution on guilt. However, the findings were not entirely consistent with this theoretical explanation, as the interaction of self-attribution and character morality was non-significant and perceived character morality had a direct impact on guilt. Future research will need to test this logic in more detail.

## Limitations

The current work has several limitations which must be addressed in future research. First, our convenience sample of young adults may limit the generalizability of our findings. It is unclear whether the effects observed here would differ for children or older adults, who may have less/more developed moral identities. Second, our findings should be replicated using other games. In the current study, the ambiguity of the stimulus allowed us to create clearly moral and immoral perceptions. Would such perceptions be possible with less ambiguous stimuli? For example, could a clear hero be portrayed as immoral and a clear villain be portrayed as moral through use of our manipulation? In a similar vein, would it be possible to alter the morality of a truly ambiguous character (e.g., a Dexter Morgan-type character who engages in evil deeds to punish those who are more evil)? Finally, consistent with feedback during the review process, some modifications were made to our statistical tests. These modifications did not alter the interpretation of findings, but they do result in the statistical model being more *post-hoc* than a priori. Thus, future research should consider our findings as tentative but promising.

## Conclusion

Video game behavior seems capable of eliciting moral emotions such as guilt. This paper explored the potential mediators of this process, particularly perceptions of character morality and attribution of in-game behaviors. The findings extend previous research and suggest future directions related to self-attribution processes. Self-attributions of virtual behavior seems to intensify guilt. Future research should continue to explore the causal mechanisms implied by theoretical models of emotion-elicitation in video game settings.

## Data Availability Statement

The raw data supporting the conclusions of this article will be made available by the authors, without undue reservation.

## Ethics Statement

The studies involving human participants were reviewed and approved by Institutional Review Board at University at Buffalo. The patients/participants provided their written informed consent to participate in this study.

## Author Contributions

All authors listed have made a substantial, direct and intellectual contribution to the work, and approved it for publication.

## Conflict of Interest

The authors declare that the research was conducted in the absence of any commercial or financial relationships that could be construed as a potential conflict of interest.
